# Mass Spectrometry of Esterified Cyclodextrins

**DOI:** 10.3390/molecules28052001

**Published:** 2023-02-21

**Authors:** Diana-Andreea Blaj, Marek Kowalczuk, Cristian Peptu

**Affiliations:** 1 Laboratory of Inorganic Polymers, “Petru Poni” Institute of Macromolecular Chemistry, Aleea Grigore Ghica Voda, 41A, 700487 Iasi, Romania; 2 Polish-Romanian Laboratory ADVAPOL, M. Curie-Skłodowska 34, 41-819 Zabrze, Poland and Aleea Grigore Ghica Voda, 41A, 700487 Iasi, Romania; 3 Centre of Polymer and Carbon Materials, Polish Academy of Sciences, M. Curie-Skłodowska 34, 41-819 Zabrze, Poland

**Keywords:** mass spectrometry, MALDI MS, ESI MS, tandem mass spectrometry, cyclodextrin, esterified cyclodextrin, polyester, cyclic esters, fatty acids

## Abstract

Cyclodextrins are cyclic oligosaccharides that have received special attention due to their cavity-based structural architecture that imbues them with outstanding properties, primarily related to their capacity to host various guest molecules, from low-molecular-mass compounds to polymers. Cyclodextrin derivatization has been always accompanied by the development of characterization methods, able to unfold complicated structures with increasing precision. One of the important leaps forward is represented by mass spectrometry techniques with soft ionization, mainly matrix-assisted laser desorption/ionization (MALDI) and electrospray ionization (ESI). In this context, esterified cyclodextrins (ECDs) benefited also from the formidable input of structural knowledge, thus allowing the understanding of the structural impact of reaction parameters on the obtained products, especially for the ring-opening oligomerization of cyclic esters. The current review envisages the common mass spectrometry approaches such as direct MALDI MS or ESI MS analysis, hyphenated liquid chromatography-mass spectrometry, and tandem mass spectrometry, employed for unraveling the structural features and particular processes associated with ECDs. Thus, the accurate description of complex architectures, advances in the gas phase fragmentation processes, assessment of secondary reactions, and reaction kinetics are discussed in addition to typical molecular mass measurements.

## 1. Introduction

**Scheme 1:**
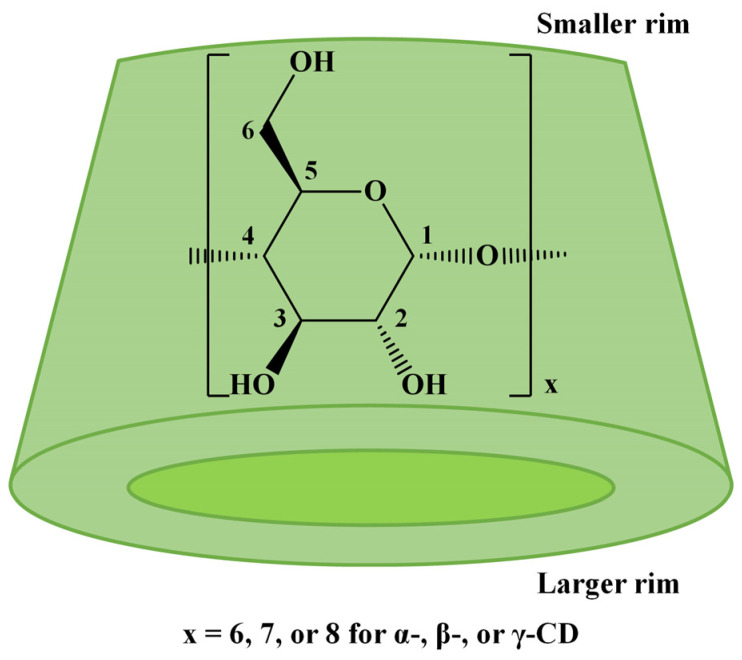
The structure of native CDs.

Native cyclodextrins (CDs) are cyclic oligosaccharides resulting from the enzymatic degradation of starch, a renewable resource [1]. The most common CDs, α-CD, β-CD, and γ-CD, consist of 6, 7, or 8 D-glucopyranose units linked by 1,4-glycosidic bonds, which lead to a conical shape with a smaller rim, displaying the primary hydroxyl groups, and a larger rim with the secondary hydroxyl groups (Scheme 1). The chemical derivatization of CDs’ hydroxyl groups is often employed to change their physicochemical properties, such as increasing water solubility or decreasing toxicity, or to change their encapsulation properties to favor the complexation of a specific guest molecule [2,3]. Generally, chemical modification of CDs may be achieved by simple reactions involving the hydroxyl groups. However, when specific properties of the modified cyclodextrins are envisaged, complicated multistep procedures are performed to achieve the targeted substitution’s regioselectivity [4] at specific OH groups. The bottleneck in CD modification arises from the presence of multiple hydroxyl functions (18, 21, or 24 for α-CD, β-CD, or γ-CD) with relatively similar reactivities (one-third are primary OH and two-thirds secondary OH).

**Scheme 2:**
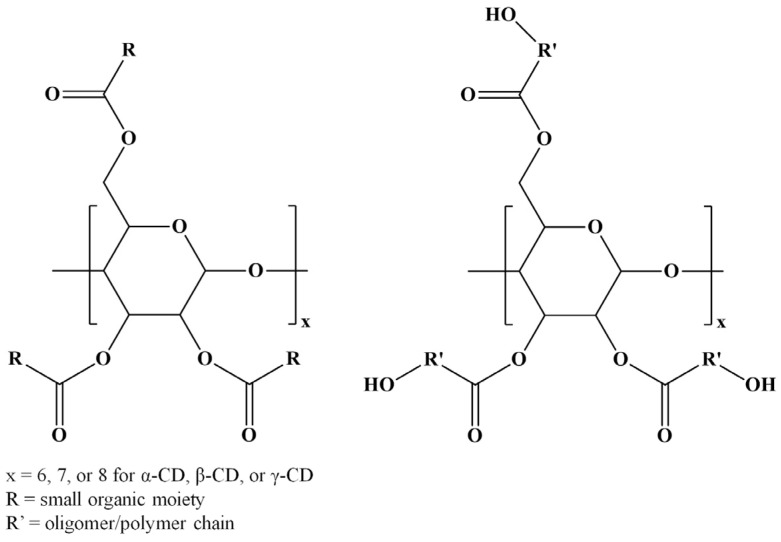
Structures of ECDs and OECD.

Among various strategies for CD chemical modification, there may be distinguished the enzyme-catalyzed transesterification reactions that lead to selective CD substitutions through ester bonds (ECD) [5]. However, several esterification reactions for CD molecules are available using various compounds, such as carboxylic acids [6,7], acyl chlorides [8,9,10,11,12,13,14,15,16], anhydrides [8,9,10,17,18,19,20,21,22,23,24,25], esters [5,26,27,28,29,30,31,32,33,34,35,36], lactones [37,38,39,40,41,42,43,44,45,46], and lactides [42,45,47,48,49,50,51]. Although regioselectivity is not always achieved in such chemical modifications, the changes in the CDs’ physicochemical properties following the derivatization procedure make the ECD derivatives attractive for various applications. Furthermore, CD molecules can be grafted with oligoester or polyester chains resulting in star-oligomer or star-polymer architectures having the CD molecule as a core. Scheme 2 presents the general structures of ECDs resulting from modification with one molecule per substitution site, or more similar molecules per substitution site, as in the case of CD-oligoesters (OECD).

Due to their particular shape, CDs have a hydrophilic outer surface and a hydrophobic inner cavity that can accommodate other hydrophobic molecules through physical interactions. The inclusion phenomena have received special attention regarding enzyme mimetics [52], for example, the phenyl ester hydrolysis as a model of chymotrypsin activity [53] or activity in the ring-opening oligomerization (ROO) of cyclic esters [37].

ECD derivatives are of particular importance due to their mix of properties like biocompatibility, biodegradability, low toxicity, and encapsulation capacity of CDs to form inclusion complexes through host–guest interactions with hydrophobic molecules [54]. The latter property is especially important in the pharmaceutical field for increasing the stability and especially the water solubility of poorly soluble drugs. Therefore, various drugs (albendazole, acyclovir, lutein, cephradine, pindolol, and amoxicillin) were encapsulated in ECDs with low substitution degrees [6,9,12,24,45,47]. On the other hand, ECDs with fatty acids lead to amphiphilic molecules that can form organized structures by self-assembly, leading to biocompatible nanoaggregates like micelles [8,9,21], spheres [9,30], vesicles [12,32], and irregularly shaped rod-like or spherical particles with matricial or lamellar structures [28]. Moreover, amphiphilic CD derivatives are expected to enhance the delivery of drugs, encapsulated in the CD cavity, in the organism [55]. Except for pharmaceutical applications, ECDs can be used as building blocks for biodegradable polymer networks [56,57], and later their ability for polymer-free electrospinning was demonstrated [58]. Furthermore, ECD carriers showed transport ability for heavy metals across polymer inclusion membranes [13] or for removing endocrine-disrupting chemicals from water [11]. ECD-based coatings for commercial polymers [14] such as polypropylene, polyethylene, polyvinyl chloride, and polyurethane were developed for industrial surface hydrophilization. Moreover, ECDs were also employed as ROP initiators [37,38,39], atom transfer radical polymerization (ATRP) initiators [15], and multiple photoinitiators and crosslinking agents when bis(acyl)phosphane oxide was attached to an ECD derivative [16].

Monitoring the derivatization of CDs is challenging because of the multiple hydroxyl groups that lead to multiple structures in the same product. The esterification of CDs through various methods can lead to derivatives with variable substitution degrees (SDs) and positional isomers with the same SD. Moreover, sample complexity may increase when oligoester chains are attached to the CD molecule, due to the molecular mass dispersity characteristic of the oligomeric substituents.

Since the development of mass spectrometry analysis with soft ionization, especially electrospray ionization (ESI) and matrix-assisted laser desorption/ionization (MALDI) [59,60], the synthesis of compounds with complex architectures, such as cyclodextrins, benefited from better structural profiling at the molecular level. In the past three decades, many native CDs and CD derivatives have been analyzed by mass spectrometry, regarding the determination of molecular masses and their distribution, the identification of attached units and end chain groups, the presence of impurities in the samples, or the formation of secondary compounds resulting from side reactions. In the case of derivatized CDs, the SD is the most important parameter, this way of describing CD derivatives being commonly used in the pharmaceutical field [61]. In this context, mass spectrometry, especially with MALDI ionization, which leads to single-charged species, is a powerful analytical tool for determining the SD of low-dispersity CD derivative samples. MALDI MS allowed facile identification of species with a certain SD as long as no mass discrimination occurs during analysis [62]. In this context, the results obtained by mass spectrometry need validation by other analytical techniques because of the lack of analytical standards for modified CDs. Nuclear magnetic resonance (NMR) spectroscopy is the most employed technique to validate the SD (or the molecular mass) obtained by mass spectrometry and also to determine the substitution site on the glycoside units, giving very good results for mono- or fully substituted CDs. However, the NMR characterization of partially substituted ECDs can sometimes be challenging due to peak broadening and lack of CD standards, but overall, the NMR technique remains the method of choice to determine the substitution site on the CD molecules. 

Although mass spectrometry can easily differentiate derivatives with various SDs, to characterize positional isomers, complementary analytical techniques must be employed, such as high-performance liquid chromatography (HPLC). For example, HPLC allows the separation of positional isomers on suitable columns, and further mass spectrometry analysis determines the exact mass of the chromatographic fractions [36]. Thin-layer chromatography was also used to confirm the formation of ECD with variable SD and to follow the progress of the esterification reaction [5,26,29,32]. Moreover, the evaluation of various synthesis systems (kinetics) may employ HPLC titration to monitor the consumption of reactants (low-molecular-mass compounds) [31]. 

The data collected by mass spectrometry characterization methods have been employed in many studies to determine the nature of substituted functions, substitution degree, molecular mass averages and dispersity, sample purity, and even to estimate the value of the hydrophilic–lipophilic balance (HLB). Moreover, offline MALDI-TOF MS (MALDI ionization with a time-of-flight mass analyzer) analysis can be employed to monitor the evolution of reaction mixtures, allowing the determination of optimum reaction conditions. Single-stage mass spectrometry studies are often validated using tandem MS fragmentation analyses by collision-induced dissociation (CID), which may lead to a structural fingerprint at the molecular level, allowing the exact identification [41]. Therefore, the current paper reviews the existing knowledge on ESI MS and MALDI-TOF MS characterization of ECD, focusing on the information brought by mass spectrometry characterization techniques for various classes of ECDs, according to their synthesis pathway. 

## 2. Single-Stage Mass Spectrometry Analysis Using MALDI and ESI

ESI MS and MALDI-TOF MS are the most employed mass spectrometry techniques for the analysis of CD-based samples. Both methods present advantages and limitations for the characterization of complex systems, such as those involving CDs. The optimum conditions for the ESI ionization technique depend on the solvent system used for the analysis of a particular sample to obtain a representative mass spectrum. ESI MS presents a major advantage in the direct analysis of sample solutions, thus making possible the hyphenation with liquid chromatography analytical techniques, such as size exclusion chromatography (SEC) and HPLC [62,63]. In principle, samples are first separated according to their molecular mass and/or to their hydrophobic/hydrophilic character and then analyzed by ESI MS. This possibility is advantageous not only due to enriching the analytical knowledge by two-stage analysis (physical separation and MS detection) but also for the analysis of samples having a certain molecular mass dispersity, such as polydisperse cyclodextrin derivatives. ESI MS has the propensity to form multiply charged species and this phenomenon is more pronounced with the increase in molecular mass. In principle, samples with narrow mass distribution lead to mass spectra where the MS peak populations with the same charge state are not overlapping, thus allowing a facile interpretation. However, wider dispersity may cause peak populations to overlap, making interpretation difficult. In this context, a prior physical separation that narrows the mass dispersity of the sample fraction entering ESI MS greatly improves the quality of the analysis.

In its turn, MALDI-TOF MS analysis is influenced by the sample preparation technique, especially by the choice of a proper matrix, compatible with the analyzed compounds, and gives mostly singly charged species that result in less complicated spectra, as compared with ESI MS [64]. Moreover, both ESI and MALDI MS analysis are limited to samples with low molecular mass dispersity because of the mass discrimination effect—lower-molecular-mass components have higher ionization efficiency and are better represented in the mass spectra as compared with higher molecular mass components of the analyzed sample. Generally, cyclodextrin derivatives have narrow molecular mass dispersity, which makes them amenable to both ionization techniques. However, when the molecular mass of the substituents is increased (e.g., oligomers or polymers) and brings a supplementary source of molecular mass dispersity (oligomers or polymers are rarely monodisperse), the total dispersity becomes significant. Thus, the distribution of molecular masses in the sample can represent a problem for ESI MS analysis, but also for MALDI-TOF MS, which is known to give accurate results for polymer samples with a dispersity lower than 1.2 [65].

MALDI-TOF MS is one of the analytical techniques which accompanied the progress in the field of carbohydrates, with studies up to 2018 being reviewed by Harvey [66,67,68,69,70,71,72,73,74,75,76]. The characterization of cyclic oligosaccharides describes various samples such as native CDs and CD derivatives; some representative examples from the literature include the study of native and methyl CDs [77,78,79,80,81,82], hydroxypropyl CDs [83,84,85,86], thiol-CD [87], sulfate-CD [88], and others. Among these, the ECDs obtained through various synthetic methods, involving multiple-step synthesis, bio-catalyzed synthesis, or ring-opening reactions, were also analyzed by MALDI-TOF MS (Table 1 and Table 2).

The instrumentation employed for MALDI-TOF MS analysis of ECD is relatively common, as the sample workup does not present special requirements for the most often performed substitution degree measurements. MALDI-TOF instruments are mentioned in the early papers, but following the instrumentation advances, MALDI TOF/TOF instruments were also used. Most of the authors applied the ECD samples on MALDI steel plates (one example used a Teflon plate [23]). Although some papers analyzed the ECD in linear positive mode [22], the positive reflector mode was used by a vast majority, especially in the most recent studies, being given the upgraded resolution. Often, the observed MS peaks in MALDI-TOF MS analysis are sodium adducts resulting either from usual impurities or by intended spiking of the sample solution with cationization agents. However, lithium, potassium, and silver ECD adducts were also analyzed, as further described. 

The most used matrices for MALDI-TOF MS analysis of native CDs and common CD derivatives were α-cyano-4-hydroxycinnamic acid (CHCA) [77,80,82,84,85,87,88] and 2,5-dihydroxybenzoic acid (DHB) [78,83,86], which is also valid for ECDs (Table 1 and Table 2). The use of trans-3-indoleacrylic acid (IAA) [20], 2′,4′,6′-trihydroxyacetophenone monohydrate (THAP) [20,23], 2-(4-hydroxyphenylazo)benzoic acid (HABA) [45], and dithranol [48] matrices may be also encountered. Polar solvents, usually used for native and modified CDs, were also employed for ECD sample analysis (water [17,36], water/acetonitrile [22,49,50,51], alcohols [5,14,27,46], chloroform/methanol [13], tetrahydrofuran (THF) [42,48], or dimethyl sulfoxide (DMSO) [31]). The analyzed samples were prepared using dried droplet or thin-layer techniques. It must be mentioned that thin-layer techniques refer either to the deposition on the MALDI MS target of a matrix solution that is left to dry, followed by the application of the analyte solution on top of the matrix layer [46,51], or to the application of a nitrocellulose/CHCA layer (prepared in acetone) that evaporates fast, followed by the deposition of the analyte solution that also contains CHCA and in some cases, trifluoroacetic acid [5,12,14,29,31].

Besides MALDI MS, a fair share of literature studies have focused on the ESI MS characterization of ECD derivatives. The mass analyzers used for electrospray ionization in ECD analysis are the single quadrupole, the triple quadrupole, the quadrupole ion trap, and the quadrupole/time-of-flight instruments. Although most of the analysis is performed in positive mode, the negative mode was also employed. Although proton-charged species are usually detected, the cation-charged ECDs are also observed. Given the deleterious presence of small amounts of salts in the laboratory glassware, ECD samples are often observed as sodium adducts, and most of the experiments employed sample spiking with salt solutions. When liquid chromatography is applied prior to the ESI source, the observed species are proton- or ammonium-charged species that are issued from the chromatographic column passage. In principle, cyclodextrins and their derivatives are rather compact and bulky molecules, with molecular masses ranging between 1000 and 3000 Da, that ionize in rather energetic conditions and have a tendency to form physical complexes in the ESI conditions with various molecules [89]. Low-molecular-weight compounds may interact with the OH groups through hydrogen bonds or may be included inside the cyclodextrin cavity. It is often possible to observe cyclodextrin clusters of two or more molecules as singly or multiply charged species that complicate the MS analysis. For such reasons, during ESI MS, significantly energetic conditions are necessary to be adjusted in order to obtain clear spectra of uniformly charged state ECD species. Therefore, capillary voltage, declustering conditions, desolvation temperature or gas flow settings, and other ESI source parameters should be adjusted accordingly.

Native and modified CDs were most often analyzed by ESI MS to determine the SD, purity of the sample, and even the positional isomers of CD derivatives by hyphenated HPLC-MS. For example, some CD derivatives characterized by ESI MS are methyl CDs [78,90,91,92,93], thiol CDs [94], or sulfate CDs [95,96]. For ESI MS analysis, most ECD samples are prepared in methanol [10,26,30,47] or water/acetonitrile mixture [41,43,49,50], but also in acetonitrile [10], methanol/isopropanol mixture [5], methanol/chloroform mixture [30], and methanol/water mixture [6].

- *MS characterization of ECD obtained through regular esterification reactions*

**Figure 1:**
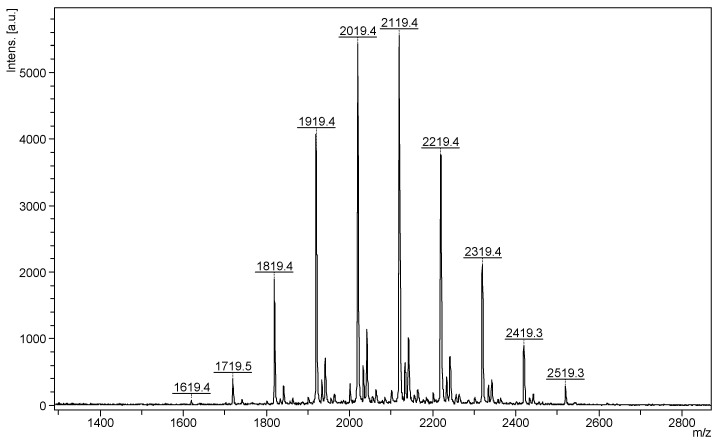
MALDI MS spectrum of succinic-anhydride-modified γ-cyclodextrin (reprinted with permission from [22]).

The most common class of ECDs that benefited from the mass spectrometry analysis may be structurally defined as acylated cyclodextrins with one molecule per substitution site, obtained through classical esterification reactions between the OH groups of cyclodextrins and carboxylic acids, acyl halogenates, or anhydrides. Such modifications of CD do not necessarily lead to regioselective substitutions, but chemical modifications alter the CD structure in such a way that the physicochemical properties of the ECD are significantly different from those of the originating CD. To the best of our knowledge, the first ECDs analyzed by MALDI MS were persuccinated derivatives of α-CD, β-CD, and γ-CD, using DHB as a matrix [17]. MALDI-TOF MS revealed the structure and the SD of the synthesized compounds. Similar studies employed the CHCA matrix to confirm the structure and the SD of partially substitute γ-CD with succinic anhydride (Figure 1). The average substitution degree determined by MALDI MS was 9.5, a close value to the one determined by potentiometric titration (9.6 succinate units) [22].

ESI MS in the positive mode was employed to study the acylation reactions of 6-O-tert-butyldimethylsilyl-β-CD using hexanoyl chloride or hexanoyl anhydride in the presence of 4-dimethylaminopyridine (DMAP) as organoactivator [8]. The mass spectrum of the derivative obtained using hexanoyl chloride showed an average substitution degree of 21 and peaks corresponding to a product having from 19 to 23 alkanoyl units; all detected species were double-charged sodium adducts. Considering that only 14 substitution sites were available, the authors concluded that secondary self-acylation reactions at the α position of acyl chains can occur, so the acylation degree could reach 28, a fact experimentally observed by ESI MS. Furthermore, ESI MS was used to establish the optimum conditions to obtain a β-CD derivative with 14 hexanoyl chains. In another study, ESI MS was used to determine the SD of acylated 6-O-tert-butyldimethylsilyl-α-, β-, and γ-CD considering the optimum conditions previously established [9]. ESI MS in the positive ion mode was also used for structural and SD confirmation of β-CD brominated at the primary hydroxyl groups and esterified at the secondary hydroxyl groups with acetyl anhydride or benzoyl bromide [10]. Mono-esterified ECD was obtained via a condensation reaction between CD and citric acid [6] using an ESI QTOF (quadrupole-time-of-flight) instrument. On the other hand, cyclodextrin modifications with maleic or itaconic [7] acids were evaluated using MALDI-TOF MS. 

Other ECD MS studies considered the structural characterization of polyfunctional initiators for ATRP synthesis of star polymers with CD cores. Thus, β-CD was esterified with 2-bromo-isobutyryl bromide and the product was carefully evaluated by MALDI-TOF MS given the fact that further polymerization depends on the exact calculation of the monomer/initiator ratio. The partial substitution was confirmed by measuring the average SD of the prepared ECD by ^1^H NMR and MALDI MS [15].

- *Characterization of ECD obtained through enzyme- and cyclodextrin-catalyzed transesterifications*

Special attention was given to the ECDs obtained through enzyme-catalyzed transesterification. Thus, the main enzymes considered in these studies were the alkaline protease [26] and thermolysin [5]. In a pioneering study using mass spectrometry for the evaluation of transesterification reactions, ESI MS (quadrupole ion trap mass analyzer) was employed to confirm the structure of monoacylated β-CD derivatives [26]. The products prepared through enzyme-catalyzed transesterification (alkaline protease from *Bacillus subtilis*) between β-CD and various divinyl dicarboxylates were analyzed in negative mode.

**Scheme 3:**
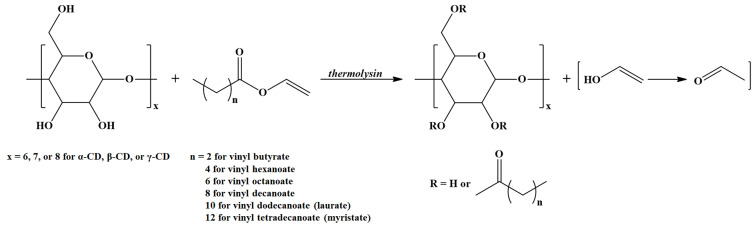
*Thermolysin*-catalyzed transesterification of vinyl esters in the presence of CDs (adapted from [31]).

A distinct class of amphiphilic ECD with potential applications for self-assembly nanostructures may be prepared via *Thermolysin* (a thermostable extracellular metalloendopeptidase from *Bacillus thermoproteolyticus*)-catalyzed transesterification of vinyl esters of fatty acids in the presence of CDs (Scheme 3) [31]. In principle, such compounds require a pinpoint calculation of the SD together with the determination of the substitution site. 

**Figure 2:**
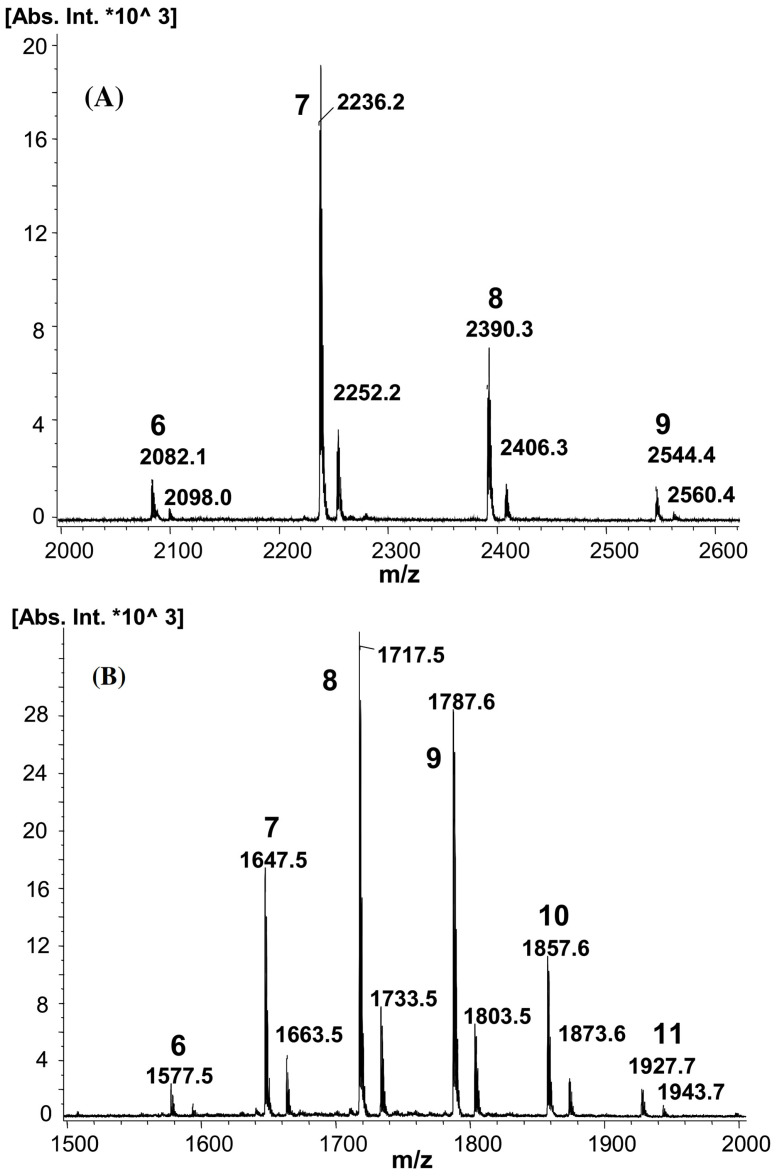
MALDI-MS spectra of β-CD decanoate esters (**A**) and β-CD butyrate esters (**B**). Masses of sodium adducts (first series) and potassium adducts (second series) are shown with numbers indicating the degree of substitution (reprinted with permission from [5]).

In a different study by Pedersen et al., the *Thermolysin*-enzyme-catalyzed reactions in DMSO led to β-CD esterification at the secondary hydroxyl groups, especially at the OH groups from position 2 as detected by NMR, for vinyl decanoate and vinyl butyrate transesterifications [5]. However, the reactions led to partial substitution at the OH groups from positions 3 and 6, more visible for β-CD-butyrate. Single-stage MALDI-TOF MS (using CHCA as a matrix) confirmed the SD of β-CD decanoate and butyrate esters (Figure 2). Based on the mass spectra of β-CD butyrate esters, an SD between 6 and 11 was calculated with the base peak corresponding to derivatives esterified with 8 butyrate units. For the β-CD decanoate esters, the SD was determined between 6 and 9, with the base peak corresponding to a derivative having 7 decanoate units. Additionally, the SD calculation by NMR and MALDI-TOF MS for the considered ECD structures showed excellent agreement. In the same study, ESI MS (direct injection using a quadrupole ion trap instrument in positive mode) was used for SD determination of α-CD, β-CD, and γ-CD laurate esters, obtained in the presence of *Thermolysin* [5].

Moreover, subtilisin-endopeptidase from *Bacillus subtilis*, alkaline serine protease *AL-89* from *Bacillus pseudofirmus,* and *Candida antarctica* lipase B, respectively, were also considered for transesterification catalysis [5]. Cyclodextrin functionalization was found to occur (following ESI MS analysis) because the of non-enzymatic catalysis process performed by the inorganic buffer salts (sodium carbonate buffer at pH 10). On the other hand, in the presence of lipase, no product was formed. 

In a similar study by Choisnard et al., the CHCA matrix was used for MALDI-TOF MS sample preparation to determine the SD of β-CD decanoate and hexanoate esters prepared through *Thermolysin* transesterification [27]. The prepared ECDs were further used for the preparation of self-organized nanoparticles with a multilamellar onion-like layered cross-section. In further work on *Thermolysin*-catalyzed reactions, MALDI-TOF MS characterization helped to determine the SD of β-CD fatty acid esters obtained from vinyl-fatty acids ester series, from vinyl-butyrate to vinyl-myristate [28].

In a more recent paper, the same group presented more proofs that support the semi-quantitative analysis of fatty-acid-modified ECD, namely α-CD, β-CD, and γ-CD decanoate derivatives, using MALDI-TOF MS, by calculating the number-average molecular mass (*Mn*) from the relative intensities of the MS peaks associated with the respective degrees of substitution [31]. The MS analysis of the synthesized amphiphilic CDs was performed using DHB as a matrix and DMSO solvent for the sample preparation. The experiments probed that the *Mn* values independently determined by MALDI-TOF MS and NMR techniques were all in agreement, also agreeing with HPLC quantitation of consumed vinyl-decanoate precursors during the reaction (reversed-phase HPLC on silica C18 column with photodiode array detection), with discrepancies under 8%. This is the first study concerning MALDI MS analysis of ECD (specific β-CD-decanoate esters) which shows that the MS method may be used in a semi-quantitative way, without significant mass discrimination. Furthermore, the authors observed that, according to the samples’ SD, the morphology of the nanoprecipitated ECD particles is changing. Thus, TEM images and SAXS analysis revealed the differences between the prepared nanoparticles with increasing SD of the ECD. Based on that, a correlation between the nanoparticles’ ultrastructure and SD was established, considering the amphiphilic character contribution to the nanoprecipitation process. Moreover, the surfactant properties of ECD were expressed as HLB (empirically established hydrophilic–lipophilic balance), as a function of the SD values calculated by MALDI-TOF MS. More specifically, the formula for determining HLB by MS was adapted after the Griffin formula [97]:

$$HLB_{MALDI}=\frac{\frac{M_{hydro}}{M_{nMALDI}^{ECD}}}{5}\times 100$$

where *M_hydro_* represents the molecular mass of the hydrophilic moiety from the ECD structure (hydrophobic alkyl chains were excluded) and *Mn_MALDI_* represents the molecular mass calculated using MALDI MS spectra.

A more complex approach made use of direct injection ESI MS and reversed-phase HPLC (silica-C18 column) coupled with evaporative light scattering detector (ELSD) or triple quadrupole ESI MS detector for the analysis of enzymatically synthesized CD fatty acid esters [30]. The considered ECDs were prepared using the previously described enzymatic transesterification catalyzed by thermolysin [27,28]. The HPLC gradient elution of ECD was performed in binary solvent systems for lower substitution degrees (acetonitrile/water or methanol/water). However, the increase in hydrophobicity, hence higher substitution degree, required ternary solvent systems (acetonitrile/acetone/water) for clearly defined and separated chromatographic peaks. Once the separation conditions were defined, the ELSD detector was replaced by ESI MS together with postcolumn flow reduction and addition of NH_4_CH_3_CO_2_ as a cation source. Automated flow injection analysis (FIA) was performed for the β-CD decanoate ester ESI MS analysis, using different injection solvents. Thus, the highest intensity signals were obtained using acetonitrile, followed by methanol and acetone. The ESI MS spectra consisted of single-charged ammonium adducts for the butyrate derivatives and sodium adducts for the other derivatives with longer chains, a fact explained by the different proton affinities and polarities. LC-MS separation revealed isobaric peaks at significantly different elution times explained by the fact that silica C18 column-ECD interactions involve only some of the fatty acid substituents. Such separation of ECD derivatives may occur because of the different placement of the substituents on the CD molecule, e.g., on the larger or on the smaller rim of the cyclodextrin molecule. Thus, positional isomers of β-CD esters (β-CD substituted at the hydroxyl groups from position 2, 3, or 6) were observed, showing the partial regioselectivity of the synthetic procedure, especially for increasing substitution degrees.

The transesterification of vinyl esters of fatty acids may be also performed in a solvent environment, only in the presence of CDs. Ladle-type monoesters of β-CD with one lauroyl group, esterified at one hydroxyl group from position 2, obtained through a self-acylating method using vinyl laurate without a base, catalyst, or enzyme, were analyzed by MALDI-TOF MS to confirm the mono-substitution of the isolated product [32].

Furthermore, m-nitrophenyl cinnamate may undergo transesterification in a basic aqueous solution containing α-CD. Thus, cinnamoyl-α-CD monosubstituted at the hydroxyl groups from position 2 or 3 were analyzed by MALDI-TOF MS, giving the expected peak at *m/z* 1125 for the sodium adduct [35]. Notably, the ESI mass spectrum (turbo ion spray source) of an equimolar mixture of 2-trans-cinnamoyl-α-CD and 3-trans-cinnamoyl-α-CD was used to confirm the formation of supramolecular clusters in aqueous solution (*m/z* from 5000 to 22,000).

MALDI-TOF MS was also used to characterize vinyl ester derivatives of α-CD, β-CD, and γ-CD synthesized using various nitrophenol esters [36] in a basic environment, according to the previously described procedure. The mass spectra of acryloyl derivatives show the presence of native CDs together with the substituted CDs. Further HPLC analysis employed a CD screen column containing 4-nitrophenyl-urea bonded to silica particles, specially designed to separate cyclodextrin mixtures through physical inclusion phenomena of the column’s stationary phase in the cavities of the eluting species. The separation was performed in a water/acetonitrile gradient with ELSD detection and MALDI-TOF MS offline analysis. The nature of detected chromatographic peaks revealed that the separation occurs according to the inclusion phenomena and, in fact, different positional isomers (MS detected) appear at different elution times. However, this was demonstrated for β-CD-vinyl esters with low substitution degrees, up to two. The increase in the SD, up to three substituents per cyclodextrin, led to fast elution (before native cyclodextrin), probably because of the interference of the crowded substituents with the inclusion-based separation on the stationary phase. Nevertheless, the analytical study confirmed that the substitution occurs at different OH groups from positions 2, 3, or 6 of the native β-CD. These results were further confirmed by LC-MS/MS experiments (vide infra Section 3). The relative SD values calculated using the MS spectra were found consistently higher as compared with those obtained via HPLC with an ELSD detector, demonstrating once more the necessary precautions when counting quantitatively the MS results.

- *Ring opening of cyclic esters*

**Scheme 4:**
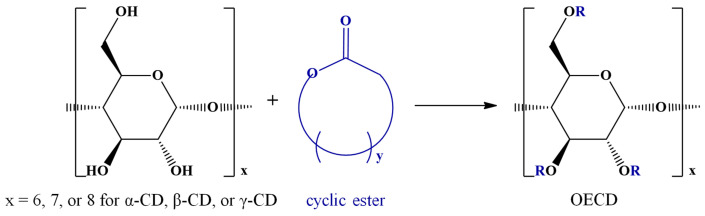
Ring-opening reaction of cyclic esters in the presence of cyclodextrins.

Cyclodextrins’ ability to form inclusion complexes with various guest molecules has been exploited for regioselective substitutions, as reviewed [98]. Moreover, the transesterification reaction of vinyl esters and nitrophenyl esters, in the presence of cyclodextrins, leads to selectively substituted ECD [99]. The selectivity of the substitution is triggered by the inclusion of the reactive species in the cyclodextrin cavity. This interesting feature related to the CD inclusion effects has been further explored by conducting ROP of cyclic esters in the presence of CDs, leading to oligoester-modified cyclodextrins (OECDs), as shown in Scheme 4. The influence of CD in such reactions was proved in the pioneering work of Harada, who showed that indeed, cyclic esters undergo transesterification reactions with CD hydroxyl groups [37]. The exact mechanism by which cyclodextrins influence such reactions is yet to be established, but the involvement of physical inclusion processes of cyclic esters into the CD cavity was unequivocally proved [38].

**Figure 3:**
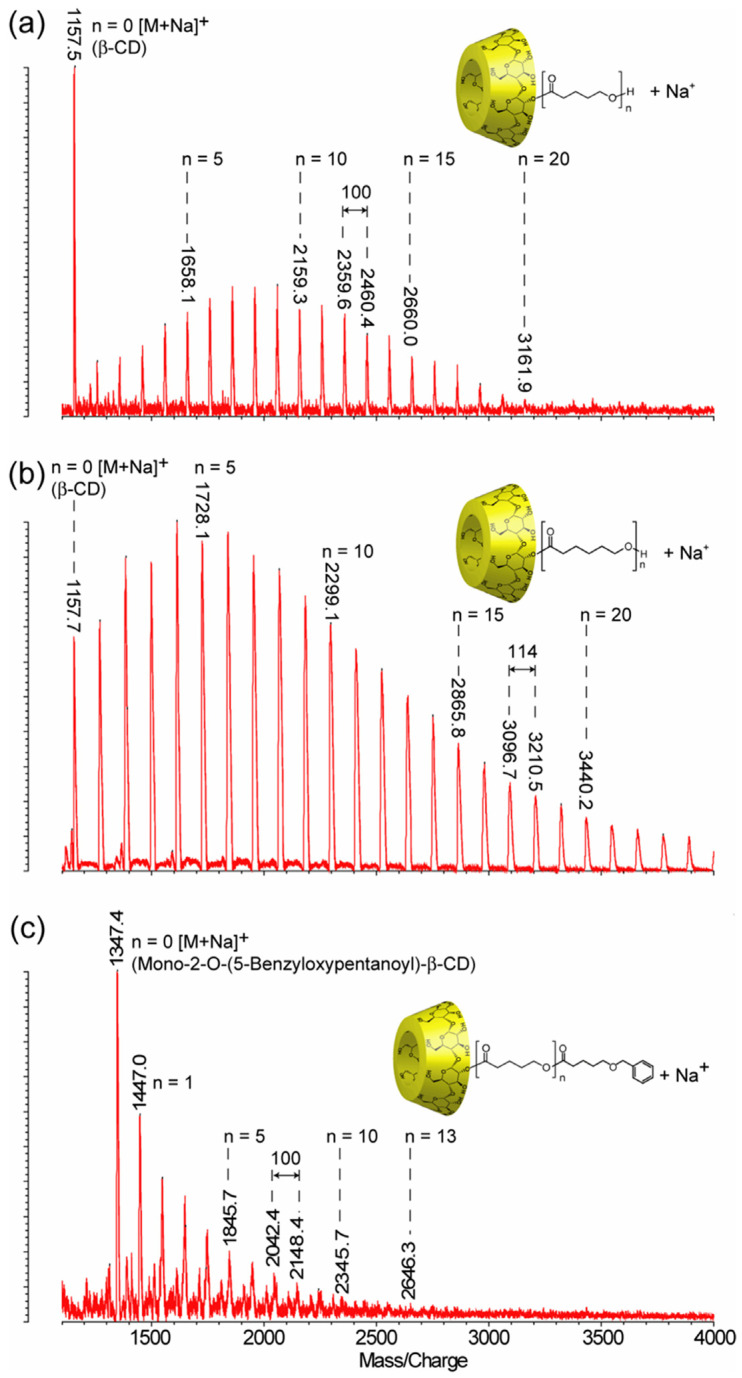
The mass spectra of ECD: (**a**) mono-2-O-poly(δ-VL)-β-CD (*m/z = 1134 (β-CD) + n* × *100 (δ-VL) + 23 (Na^+^))*, (**b**) mono-2-O-poly(ε-CL)-β-CD (*m/z = 1134 (β-CD) + n* × *114 (ε-CL) + 23 (Na^+^))*, and (**c**) mono-2-O-(5-benzyloxypentanoyl)-poly(δ-VL)-β-CD (*m/z = 1324 (mono-2-O-(6-benzoxy-pentanoyl)-β-CD) + n* × *100 (δ-VL) + 23 (Na^+^))* (reprinted with permission from [38]).

MALDI-TOF MS was used together with NMR spectroscopy to thoroughly investigate the ring-opening reaction of cyclic esters initiated by CDs. The first ring-opening polymerization of lactones (β-butyrolactone (β-BL), δ-valerolactone (δ-VL), ε-caprolactone (ε-CL)) initiated by α-CD, β-CD, and γ-CD was performed in bulk polymerization conditions, and the products were characterized by MALDI-TOF MS, using DHB as a matrix, to have a qualitative proof for the synthesis of polyester-tethered CDs [37,38,39]. The mass spectra consisted of peak series with peak-to-peak differences specific to the lactone monomer unit (86 Da for β-BL, 100 Da for δ-VL, and 114 Da for ε-CL). For example, the qualitative evaluation of a representative mass spectrum of modified β-CD with poly(δ-VL) or poly(ε-CL) may be reasoned using the respective equations *m/z(VL) = 1134 (β-CD) + n* × *100 (δ-VL) + 23 (Na^+^) (*Figure 3a) or *m/z(CL) = 1134 (β-CD) + n* × *114 (ε-CL) + 23 (Na^+^)* (Figure 3b) [38].

Other ECDs (mono-2-O-(6-benzoxy-pentanoyl)-β-CD [37,38,39] and mono-cinnamoyl-α-CD [38]) were also used as initiators for the ring opening of δ-VL. MALDI-TOF MS analysis revealed the formation of the poly(δ-VL)-ECD containing the modified cyclodextrin initiators (Figure 3c), thus confirming their capacity to initiate the reactions.

**Figure 4:**
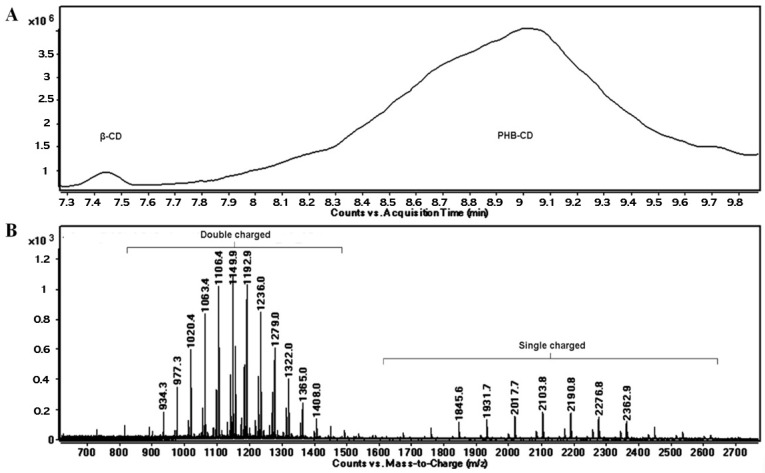
Total ion chromatogram (**A**) and LC-MS spectrum of PHB-CD derivatives (**B**) (reprinted with permission from [41]).

The OH functions of cyclodextrins may initiate the ring opening of cyclic esters in a regular manner, similar to other OH functional derivatives, using classical organometallic catalysts [100] and organic activators [101]. The amine-activated transesterifications of cyclic esters in the presence of cyclodextrins were performed and characterized by reversed-phase gradient elution LC-MS (LC QTOF instrument), using a silica C18 column [41]. Thus, structural characterization of oligo(3-hydroxybutyrate)-β-CD (PHB-CD) derivatives, obtained by the ring opening of β-BL in the presence of β-CD and (-)-sparteine as an organic activator, in bulk, revealed that free β-CD, PHB homopolymers, and CDs modified with 3-hydroxybutyrate were obtained in the reaction mixture. PHB-CD was evaluated by LC-ESI MS and not by direct infusion in the ESI source to reduce the ion suppression effect exerted by the simultaneous injection of oligomer species with significantly different molecular masses (Figure 4).

In this chromatographic separation, the oligo(3-hydroxybutyrate)-β-CD derivatives were separated according to the number of monomer units attached to the cyclodextrin, as previously observed for other ECD [30,36]. Although the ECD species with increasing substitution degrees were still overlapping in the LC-MS chromatogram, the obtained extracted ESI MS spectra showed an improvement over the direct injection analysis, allowing the identification of the ECD product. The separation method was less capable of differentiating different substitution degrees of ECD, as in the case of ECD with fatty acids [30], because of the differences between the hydrophobic character of the substituents. The average substitution degree of PHB-CD derivatives, detected by LC ESI MS, was found to be 14 monomer units per cyclodextrin molecule, calculated on the peak series corresponding to the double-charged species of higher intensities. However, ^1^H NMR analysis revealed that the average length of the PHB oligoester chains attached to the cyclodextrin is about 3 monomer units. This result, together with the average number of monomer units per CD obtained from the MS analysis, demonstrates that the obtained PHB-CD derivatives are functionalized with multiple oligoester short chains.

Moreover, two minor populations of PHB-CD derivatives were detected, with one or two crotonate units as end groups. The presence of the crotonates in their structure was reflected by the longer elution times as compared with the main PHB-CD population because of their decreased polarity. The base peaks of the secondary β-CD derivatives detected in the mass spectra corresponded to 13 and 12 hydroxybutyrate units with one and two crotonate groups, respectively. Assuming similar ionization efficiency, a ratio of 1:0.4:0.08 was calculated between the three PHB-CD derivatives, based on their relative intensities in the extracted LC-MS spectrum. These species were not confirmed by NMR analysis because of their high dilution in the analyzed mixture.

In a later study, the synthesis of structurally similar derivatives was performed by conducting the ring-opening reaction of β-butyrolactone in the presence of β-CD and (-)-sparteine in a DMSO solution [43]. In contrast with the bulk reaction, unreacted β-CD was not detected in the LC-MS analysis, although species with crotonate groups were consistently present as double-charged adducts with sodium and ammonium. The LC-MS characterization showed that cyclodextrins are homogeneously functionalized with an average of 4 monomer units per cyclodextrin molecule. Complementary NMR spectroscopy analysis showed that the monomer units are distributed as 3-OH butyric acid among different glycoside units, at C6 positions.

ECDs with 3-hydroxy-butyrate arms were also prepared via ROP of β-BL in the presence of an organometallic catalyst and using a bis-hydroxyl functional β-CD (dihydroxyl per-O-benzyl-β-CD), enabling the obtention a two-arm structure [42]. MALDI-TOF MS (using CHCA matrix) was employed to qualitatively describe the PHB polymers. Thus, MS peaks in the region of 8000 *m/z* were assigned to two-arm ECDs having over 64 monomer units. In addition, water-initiated PHB homopolymers were identified in the low-mass region of the MALDI MS spectrum.

Polylactide (PLA) represents another type of biodegradable oligoester that may be attached to cyclodextrins through the ring opening of lactide monomers. One of the early works that took advantage of the possibility to obtain water-soluble ECD derivatives through ROO of lactide (LA), without any catalysts, used ESI MS (triple quadrupole mass analyzer) in positive mode to characterize the product [47]. The structural confirmation was based on NMR characterization and also on the MS analysis. The ESI MS spectrum of the final product presented one main peak series corresponding to β-CD-oligolactide (CDLA) species, having the base peak associated with an ECD product containing 6 lactate monomer units. Later studies used MALDI-TOF/TOF MS (CHCA matrix) to characterize the ECD product issued from the ring-opening oligomerization (ROO) of L-lactide in the presence of β-CD, performed in N,N-dimethylformamide (DMF) solution [50]. The calculations performed by taking into consideration the relative intensities of the MS peaks revealed that an average of 15.6 lactate units were attached to β-CD. The observed main peak series was assigned to even-numbered oligolactates, but species with odd numbers of lactate units were also detected as a minor peak series, showing that transesterification reactions occur in this reaction system.

**Figure 5:**
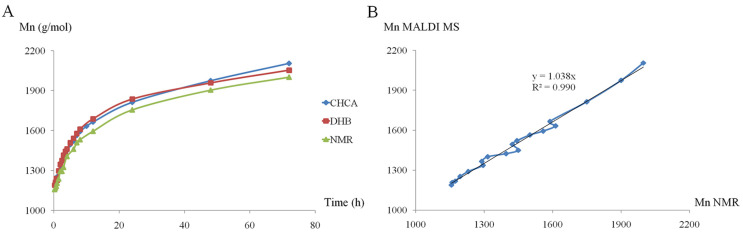
Mn (number-average molecular mass) evolution of β-CDLA product determined by ^1^H NMR and MALDI MS (DHB—dried droplet, CHCA—thin-layer) (**A**) and the agreement between the *Mn* evolution determined by ^1^H NMR and CHCA (**B**) (reprinted with permission from [51]).

In a more recent study, MALDI-TOF MS (on a TOF/TOF mass analyzer) was employed to monitor the minute changes in *Mn* evolution for the ROO of D,L-lactide in the presence of β-CD, performed in different reaction conditions (with the following reaction parameters: solvents, temperatures, β-CD/lactide molar ratios, concentrations, and even the effect of a common ROP organo-activator, DMAP) [51]. To accurately reflect the evolution of the reaction mixture content and to provide a semi-quantitative description of the reaction system, the MALDI-TOF MS method needs validation by other methods. Previously, the kinetics performed for the ROP of lactide, initiated by (+)-sparteine, were studied by MALDI-TOF MS (MALDI QTOF mass spectrometer) and SEC, finding a satisfactory agreement between the two methods [102]. The values of the molecular mass determined by SEC were 1.5 times higher than those determined by MALDI-TOF MS, but the MS analysis accurately described the general trend regarding the monomer conversion rate. Therefore, to evaluate the ROO kinetics in the presence of β-CD performed in DMF (a reaction system similar to previous works [47,50]), the authors employed ^1^H NMR spectroscopy for the validation of the results obtained by MALDI-TOF MS (Figure 5) [51].

As observed in the plot from Figure 5A, the evolution of the *Mn* (calculated using MALDI MS spectra) was followed by taking into consideration two matrices, CHCA and DHB, and comparing with lactide monomer conversion followed by ^1^H NMR spectroscopy. Thus, based on the agreement between NMR and CHCA preparation (Figure 5B), the authors selected this MALDI sample preparation for further kinetics experiments involving β-CDLA synthesis. Thus, MALDI MS kinetics witnessed the specific reactivities, for the considered reaction systems, induced by the changes in the reaction conditions. For example, the increase in temperature led to higher system reactivity and higher values of molecular masses, but it was also revealed that depolymerization reactions can occur at higher temperatures and longer reaction times. Moreover, the NMR investigation (Distortionless Enhancement by Polarization Transfer—DEPT-135 experiment commonly used to determine the multiplicity of carbon atoms) of the β-CDLA product revealed the selective substitution at the primary hydroxyl groups of the β-CD molecule. Similar substitution patterns were also determined for β-CDLA products obtained in bulk [49].

**Figure 6:**
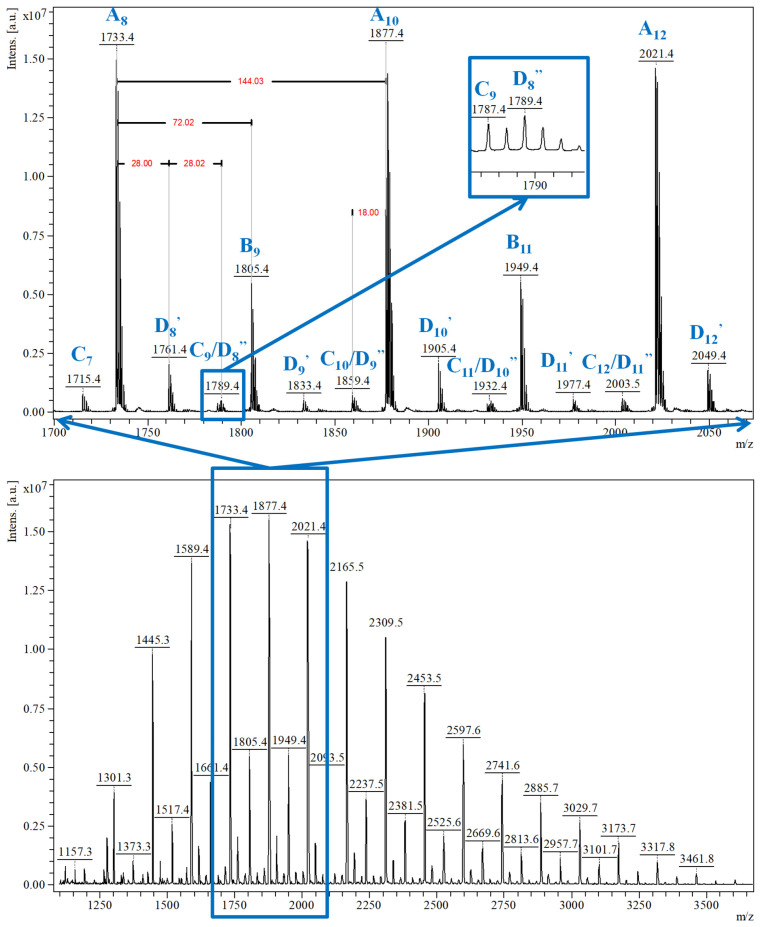
MALDI MS spectrum of CDLA derivative showing the MS peak series detected in the CDLA sample (reprinted with permission from [51]).

Qualitative MALDI-TOF MS characterization (Figure 6) revealed that two main peaks series are formed: A series of peaks with higher relative intensity, associated with the main CDLA product, and B series assigned to the CDLA resulting from transesterification reactions. Based on the high degree of structural similarity and under the provision that the ionization efficiency is similar for β-CDLA derivatives from A and B series, the authors assumed a semiquantitative relationship and calculated the percent of the B series from the A series to determine the propensity of transesterifications in reaction systems performed at different temperatures, thus showing the transesterification increase with temperature. A third series, annotated as the C series, revealed that water elimination processes may also occur. More importantly, MALDI MS characterization showed that solvent degradation side-reactions can occur, in DMF and N-methyl-2-pyrrolidone (NMP) systems, which lead to an overall increased system reactivity, but also to structural changes. Thus, the MS peaks were associated with the attachment of DMF-derived formate organic moieties (noted as D series—Figure 6, highlight) or NMP-derived moieties (noted as E series) to CDLA derivatives. Such a thorough MALDI MS study demonstrates that the proper reaction conditions can be determined to favor the formation of the CDLA derivatives with desired molecular mass while minimizing the propensity of the secondary reactions.

In another study, MALDI-TOF MS (with dithranol as matrix) was employed to characterize the β-CD diol end-capped PLA, obtained with DMAP as an organo-activator [48]. In this case, the two types of MS peak populations were also detected as silver adducts (silver trifluoromethanesulfonate was used as a cationization agent). Furthermore, MALDI MS structural characterization was used to describe the products obtained through the ROP of rac-LA initiated by dihydroxyl per-O-benzyl-β-CD (β-CD diol) in the presence of an organometallic catalyst [42].

MALDI-TOF MS characterization (on a TOF/TOF mass analyzer) provided valuable insights on the bulk polymerization of L-lactide performed only in the presence of cyclodextrins (α-, β-, and γ-CD), qualitatively describing the reaction products containing unreacted CDs, CDLA conjugates with different substitution degrees, and PLA homopolymers [49]. The reaction was performed using excess L-lactide monomer vs. cyclodextrin initiator to ensure homogeneous mixing conditions in the melt monomer environment. Given the complex mixture, it is difficult to quantify the resulting products using direct MS measurements because of mass discrimination effects and the relatively different ionization efficiency between neat and modified CDs. Previous works employed MALDI MS qualitative analysis of purified products in THF [37], and in a first attempt, the authors fractionated the reaction mixture in THF and performed the MALDI MS measurement of the resulting fractions for each α-CDLA, β-CDLA, and γ-CDLA derivative [49]. The MALDI MS analysis of THF-soluble products revealed average substitution degrees of 26 in the case of α-CD and 28 for γ-CD, while β-CD led to a higher substitution degree of 36 lactate monomer units, which allowed a performance evaluation of the employed CDs in the ROP of L-LA.

**Figure 7:**
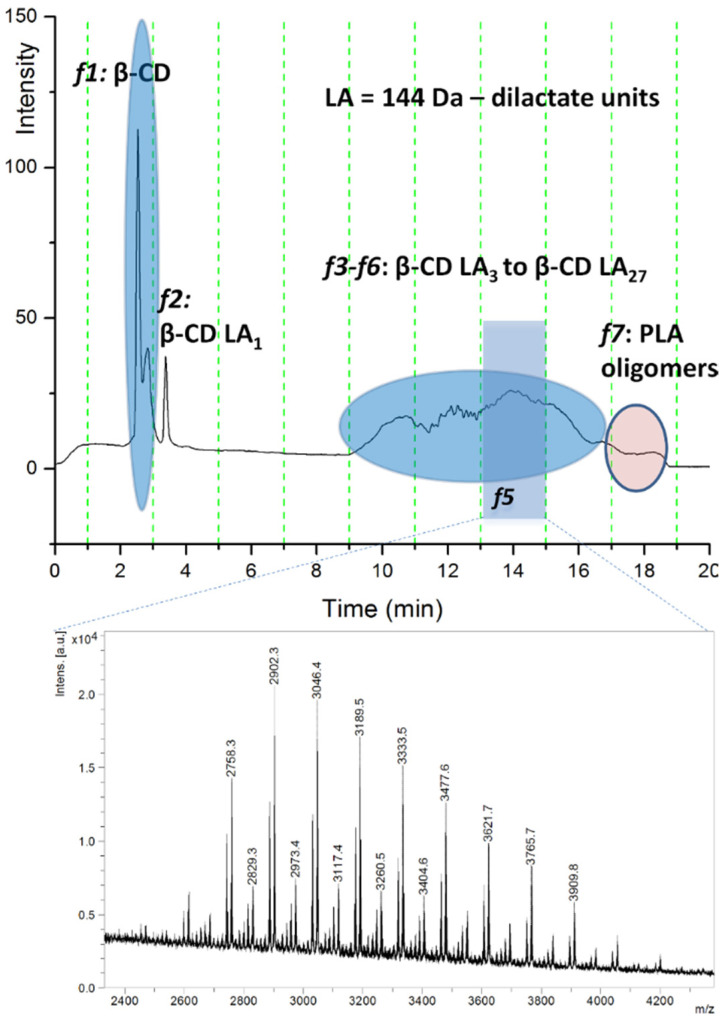
ELSD chromatogram of crude β-CDLA mixture and MALDI MS spectrum (reprinted with permission from [49]).

On the other hand, the quantification of the unreacted cyclodextrin content in these systems was directly correlated with their performance in the ring-opening process. Further experiments involved deeper characterization through HPLC chromatographic separation (reversed-phase gradient elution separation on a silica-C18 column) with online ELSD detection and offline MALDI MS analysis. The eluted compounds were separated according to the increasing number of lactate monomer units attached to the β-CD. Thus, the chromatogram exemplified in Figure 7 describes the relative content of reacted β-CDLA and native β-CD in the β-CDLA reaction mixture, as measured by ELSD detection, in parallel with MALDI-TOF MS chromatographic peak identification (TOF/TOF mass analyzer).

Based on similar experiments, the authors calculated and compared the amounts of free CD in the employed reaction systems for all three CDs, α-, β-, and γ-CD, respectively, using the integration of the HPLC ELSD chromatograms. The obtained results revealed that for the ROP process of L-LA in bulk, γ-CD is less active as compared with α-CD and β-CD, thus demonstrating a certain correlation between the cavity size and the employed cyclic ester for the bulk polymerization process. Moreover, species with an odd number of lactate units were also detected in the mass spectra of all high-molecular-mass derivatives, together with some PLA homopolymers in the region of lower molecular masses. LC-ESI MS (gradient reversed-phase separation on a C18 silica column and detection on an ESI QTOF detector) was also employed to characterize these CDLA derivatives. However, double- and triple-charged species were detected in the case of β-CDLA, increasing the complexity of the mass spectrum; thus, finally, MALDI MS analysis was preferred.

Bulk ROO of ε-CL in the presence of dry or wet β-CD and under an inert atmosphere at high pressures was also investigated [44]. Two products were detected in the MALDI-TOF MS analysis, one corresponding to β-CD-oligocaprolactone (β-CDCL) and a second one to water-initiated polycaprolactone (PCL) homopolymers. Unfortunately, MS spectra were further employed to calculate the relative content of β-CD-based derivatives and PCL homopolymers without considering the differences in ionization efficiency given by the mass discrimination effect and also by the different chemical nature between β-CDCL and PCL homopolymers. Such evaluations should make use of chromatographic separation with non-discriminative detections between the two species, for example, ELSD [49]. Based on the conducted experiments, the authors concluded that β-CD can act similarly to an artificial lipase, leading to the polymerization of ε-CL by monomer inclusion in the inner cavity, which promotes cyclic ester hydrolysis [44]. Moreover, MALDI-TOF MS was also employed to study the transesterification of PCL in the presence of β-CD. The mass spectra for the reaction performed in the presence of β-CD showed that after 24 h, the PCL homopolymer content decreases, while the β-CD-oligocaprolactone content increases due to transesterification reactions. Macrocyclic oligocaprolactone species with 6 to 13 units were also detected in the mass spectra.

In a recent study, MALDI-TOF MS (TOF/TOF mass analyzer) was employed to determine the evolution of molecular masses and the identification of products’ structures resulting in solution ring-opening oligomerization of ε-CL using β-CD as an initiator [46]. The employed MS method enabled the semi-quantitative evaluation of the products, thus comparing the influence of different reaction parameters (DMF and DMSO solvents and the presence of organo-activators (DMAP and (-)-sparteine)).

**Figure 8:**
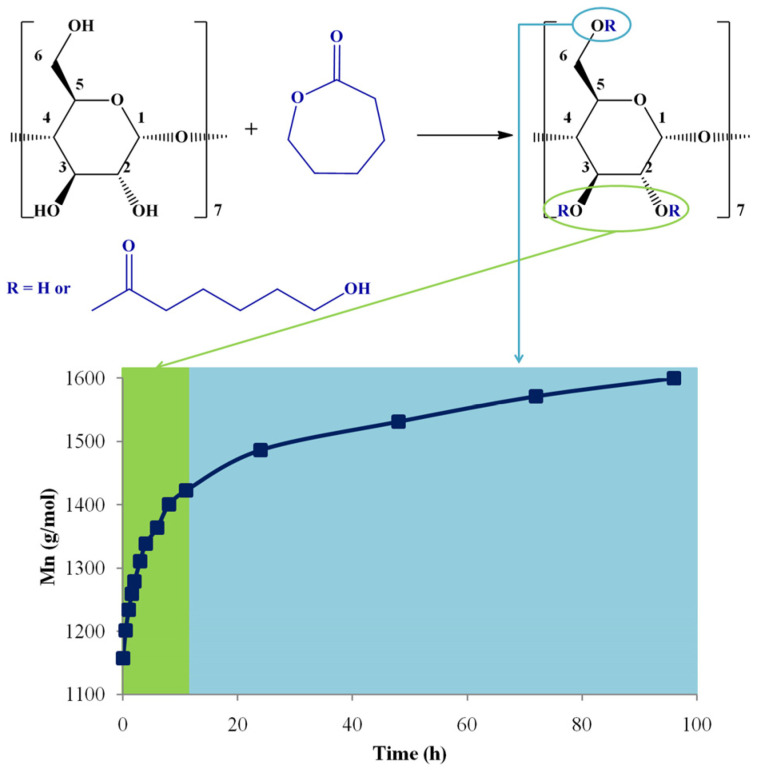
The substitution pattern evolution of CDCL derivatives with Mn increase (reprinted with permission from [46]).

The MALDI-TOF MS evaluation method, monitoring the *Mn* increase, was validated by calculating the equivalent ε-CL monomer conversion, using ^1^H NMR spectroscopy. The method optimization took into consideration two MALDI preparations, using CHCA and DHB matrices, respectively. Each *Mn* determination by MS was compared with the equivalent ε-CL conversion, measured by ^1^H NMR spectroscopy, assuming that the only process responsible for the monomer consumption is the transformation in the CDCL products. Therefore, for the ε-CL ring-opening reaction in the presence of β-CD, performed in DMSO and with the addition of DMAP, a very good agreement between NMR and MS methods was established using the DHB matrix. Although the CHCA matrix led to a slight overestimation of the molecular mass values, the *Mn* evolution trend was similar. Moreover, the reaction kinetics performed by NMR spectroscopy allowed the in-depth investigation of the substitution pattern (Figure 8). At the beginning of the reaction, the substitution of β-CD occurs at the secondary hydroxyl groups (OH2, OH3). After about 12 h of reaction time, the modification of β-CD with caprolactone units occurs also at the primary hydroxyl groups (OH6).

**Figure 9:**
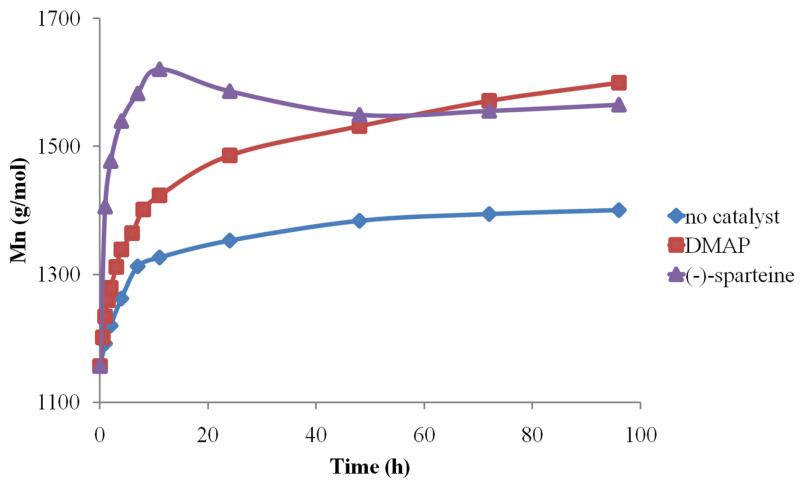
Organo-activator influence on CDCL *Mn* evolution (reprinted with permission from [46]).

After establishing the MALDI-TOF MS analysis conditions, the method was further used to determine the organo-activator influence in both solvents. Therefore, the MALDI MS data concerning the *Mn* evolution revealed that (-)-sparteine systems are the most active, followed by DMAP and finally by pure solvents (Figure 9).

**Figure 10:**
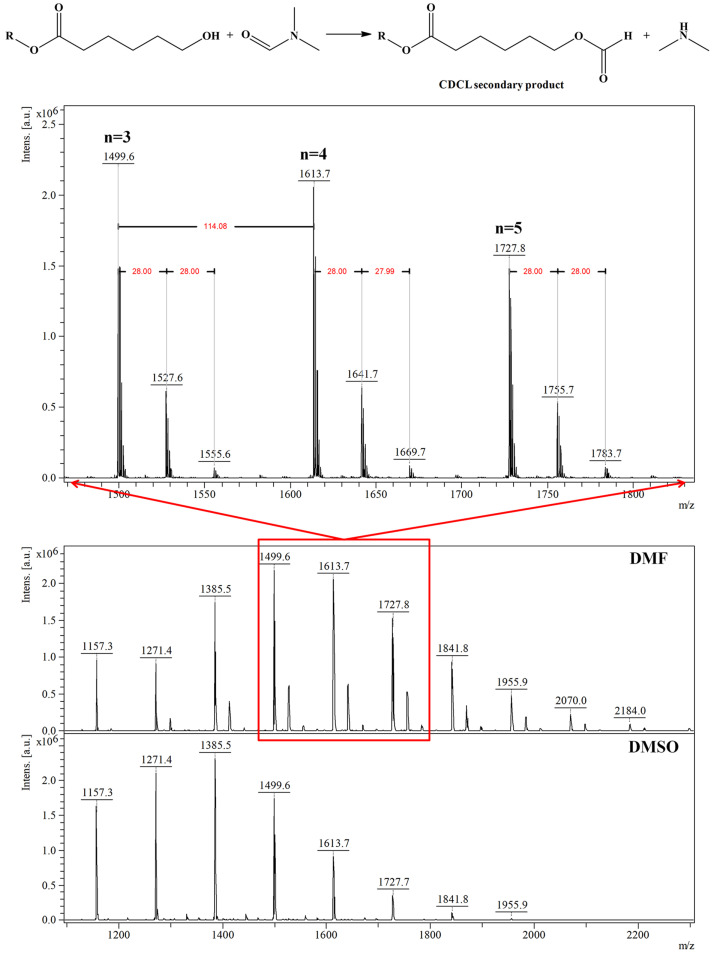
MALDI MS spectrum of CDCL derivatives obtained in DMF or DMSO and DMF degradation reaction in the presence of CDCL (reprinted with permission from [46]).

Depolymerization reactions also occurred in the presence of (-)-sparteine, signaled by the observed decreases in *Mn* values. Depolymerization reactions also led to higher molecular mass dispersity of the samples, as determined by MS, while the use of DMAP organo-activator led to lower dispersity values, even at longer reaction times. Moreover, the results obtained by MALDI-TOF MS showed that DMF systems are more active than DMSO because of solvent degradation during the reaction, which leads to dimethylamine formation and also to the attachment of formate moieties to CDCL derivatives, detected as MS peaks upshifted with 28 Da from the main series (Figure 10—MS spectra and the formation of secondary products). The amine secondary product is acting as an additional organo-activator, thus explaining the higher polymerization rates observed for DMF solvent.

## 3. Multistage Mass Spectrometry Analysis

Native CDs, their derivatives, and inclusion complexes are structurally diverse, and mass spectrometry represents a powerful tool for the elucidation of their chemical structure. Although many studies employed mass spectrometry techniques for the characterization of CD-based compounds, only a few studies used tandem mass spectrometry to perform structural identification based on fragmentation patterns [93,103,104,105,106,107,108,109,110,111,112,113]. The fragmentation patterns of CD-based structures can be influenced by the structure of the monomer units (substituted and unsubstituted glycoside units), the nature of the substituent groups (small molecules or polymeric chains), the binding affinity towards certain (metal) ions, and the presence of guest molecules physically included in the CD cavity [112]. ECDs may be assimilated as block oligomers that undergo fragmentation pathways related to the cleavage of chemical bonds situated in the oligosaccharide block or to the cleavage of attached moieties through ester bonds [41,50,114].

Linear oligosaccharide fragmentations are generally described using the nomenclature for the fragment ions proposed in the work of Domon and Costello [115]. Linear oligosaccharides have a non-reducing end, which gives fragment ions named A, B, and C, and a reducing end which gives X, Y, and Z fragment ions. The pairs of B/Y and C/Z fragment ions form due to the cleavage of the semiacetalic bonds, while the pairs A/X result from the cross-ring cleavage of the glycoside unit. However, cyclic oligosaccharides do not have non-reducing and reducing ends; thus, the nomenclature of fragment ions is adapted.

The fragmentation of various cation-charged cyclodextrin parent ions proceeds mainly via the cleavage of semiacetalic bonds, leading to consecutive losses of 162 Da [105,106,107,110], corresponding to one glycoside unit, but losses of 264 Da were also identified for double-charged species due to cross-ring fragmentation [107].

**Scheme 5:**
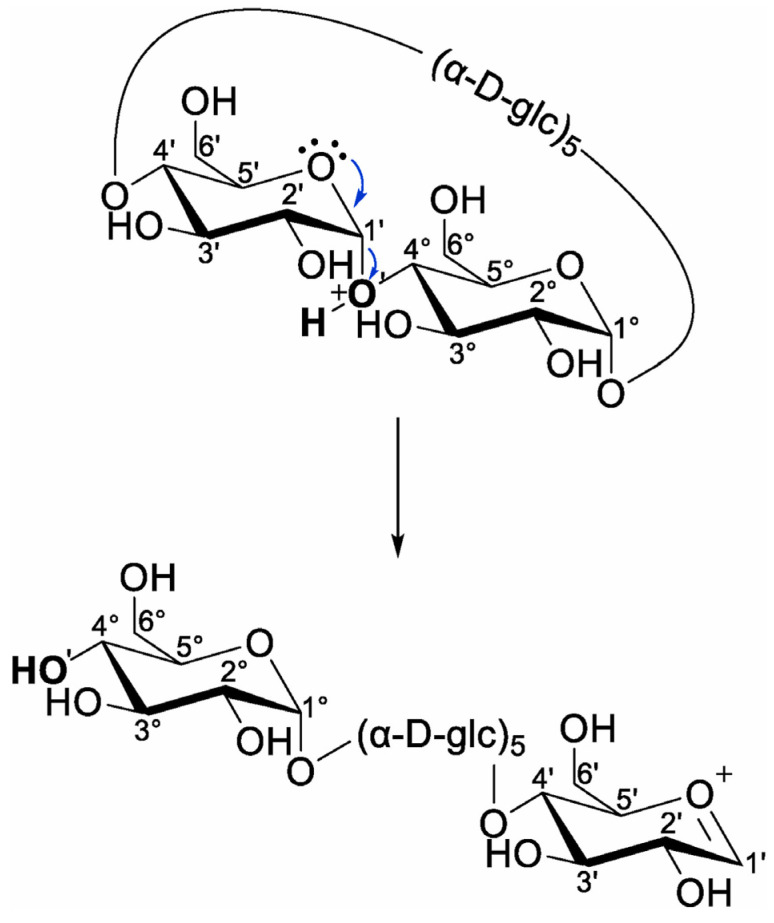
Charge-induced ring opening and linearization of proton-charged β-CD, resulting in the formation of a new non-reducing end (reprinted with permission from [112]).

In their recent work, Bruni and Schurch studied the fragmentation of proton-charged β-CD through ion trap CID and higher-energy C-trap dissociation on a hybrid mass spectrometer that combines a linear ion trap and the Orbitrap mass analyzer. They established that the fragmentation onsets with the ring opening of the β-CD macrocycle, leading to a linearization of the cyclic oligosaccharide (Scheme 5), followed by consecutive neutral losses of glycoside unit through a charge-independent mechanism because the charging proton is extracted during the macrocyclic cleavage, being unavailable for subsequent bond cleavages [112]. As a novel hydroxyl unit results from linearization, the nomenclature used for linear oligosaccharides can also be applied to cyclic oligosaccharides.

**Scheme 6:**
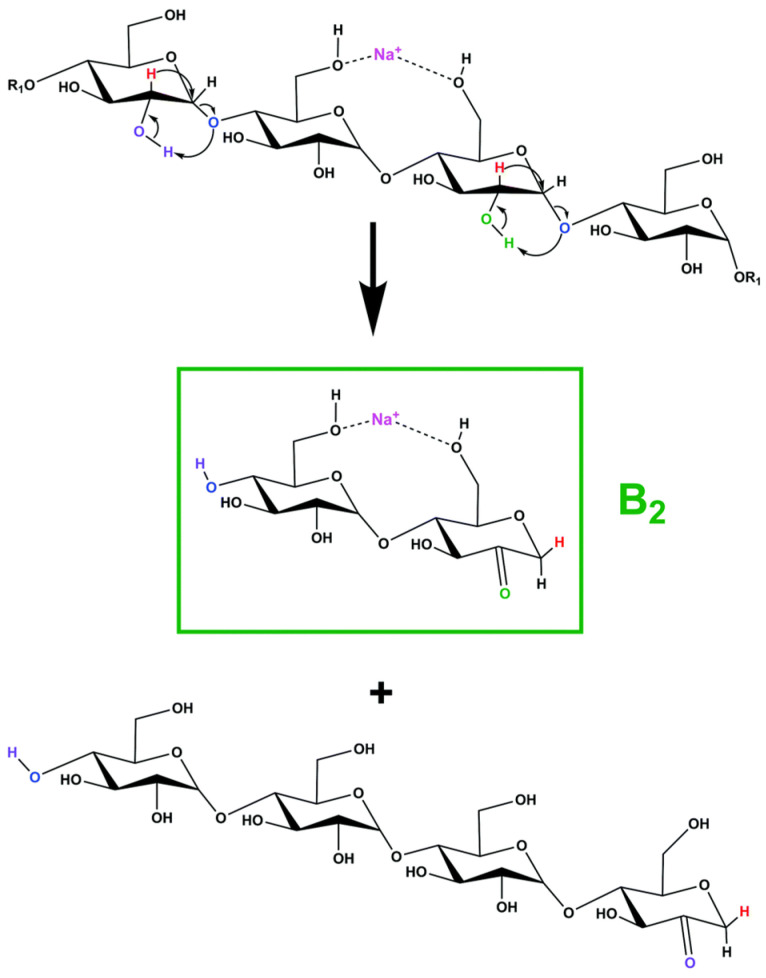
Proposed fragmentation mechanism for the formation of the fragment with 2 glycoside units (reprinted with permission from [113]).

Another recent study concerning the fragmentation of sodium-charged β-CD employed a homebuilt high-resolution ion mobility mass spectrometer with cryogenic infrared action spectroscopy to obtain FTIR cryogenic spectra of specific fragment ions resulting from CID fragmentation [113]. Simulation studies associated with the cryogenic FTIR data, regarding the main sodium-charged fragments, allowed the structural identification of the corresponding structures. Their results indicated that one type of structure is energetically favorable to be formed during β-CD fragmentation, the 2-ketone-type structure, as shown in Scheme 6. The experiments and simulation studies also revealed that in the gas phase, only one sodium-charged conformer is stable for fragments with 2, 3, 4, and 5 glycoside units, while for the fragment corresponding to 6 glycoside units, more than one stable conformer can be formed.

**Scheme 7:**
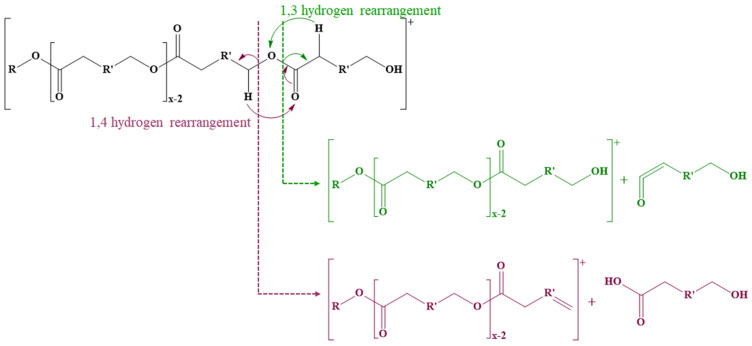
Fragmentation of polyesters on the acyl or alkyl part of the ester bond.

The fragmentation of ECD also needs to consider the general fragmentation pathways characteristic of polyesters. Thus, the cleavage of ester bonds leads to three main fragmentation pathways (Scheme 7) [116,117,118,119]. The first mechanism involves the cleavage of ester bonds through 1,4-hydrogen (McLafferty) rearrangements when the 1,4-hydrogen is transferred to the oxygen from the carbonyl group leading to the formation of two compounds, one with a carboxyl end group and one with an unsaturated end chain. The second fragmentation mechanism takes place through 1,3-hydrogen rearrangements when hydrogen from the methylene group from the α-position is transferred to the ether oxygen of the ester bond, leading to the formation of a compound with an unsaturated carbonyl end group (a carboxylic acid group without a water molecule) and an alcohol. Intramolecular transesterification reactions may also occur, similar to backbiting processes; an alcohol end group is required for this mechanism. The oxygen from the hydroxyl group at the end of the polyester chain performs a nucleophilic attack on carbon from the ester carbonyl group, leading to the formation of a macrocyclic ester and an alcohol.

Based on the typical fragmentation patterns associated with the structural components of ECD, a structural identification at the molecular level may be performed for various ECD structures. In the first example, LC-MS/MS analysis was employed as an additional technique to MALDI MS and HPLC ELSD for structural confirmation of ECD [36]. The fingerprint of the acrylated cyclodextrin derivatives was established using the combination between the CD Screen LC separation and CID fragmentation of the resulting chromatographic peaks on an LC-QTOF MS analyzer setup. For example, the monosubstituted β-CD-acrylate was chromatographically separated into two peaks which were characterized by different MS/MS patterns (caused by different positional isomers with different collisional stability—substitution at the primary or secondary OH groups). The authors concluded that the chromatographic peaks observed at different elution times are positional isomers resulting from synthesis, thus demonstrating the lack of regioselectivity in the substitution. The α-CD acrylate derivative led to similar results, although the separation of pure β-CD from the derivatives was not complete. MS/MS data could not be obtained for the undecenoyl β-CD derivative, having one peak from the monosubstituted derivative partially overlapped on the pure β-CD, in the extracted ion chromatogram.

CID MS/MS analysis and multi-stage MS^3^ experiments using a hybrid triple quadrupole-linear ion trap mass analyzer were performed on gemini surfactants based on β-CD substituted with N,N-bis(dimethylalkyl)-α,ω-aminoalkane -diammonium moieties (alkyl = hexadecyl, dodecyl, or oleyl) through succinyl spacers to establish fragmentation patterns for further LC-MS/MS analysis [25]. The fragmentation occurs similarly for all derivatives and onsets with the loss of one quaternary ammonium head group along with the attached alkyl tail, followed by the loss of one glycosyl unit. Furthermore, two fragmentation pathways were identified. The first pathway consists of consecutive losses of glycosyl residues and alkyl tails, while in the second pathway, the loss of the other ammonium head group along with the attached alkyl tail occurs first, followed by the loss of dehydro-glycosyl units. Moreover, during the fragmentation experiment, the cleavage of the ester bond that links the substituent to β-CD does not occur.

**Scheme 8:**
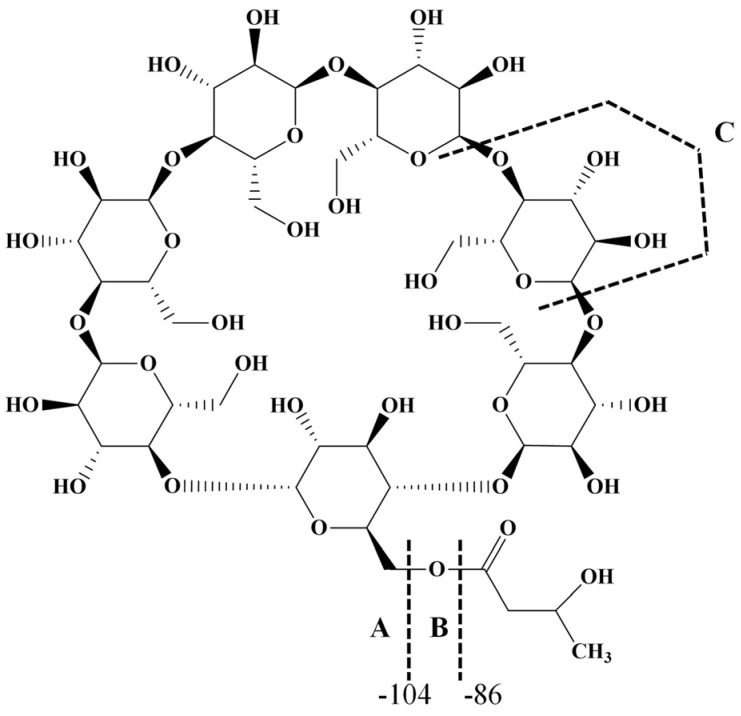
Fragmentation pathways of PHB-CD (adapted from [114]).

ESI CID fragmentation studies of PHB-based ECD derivatives [41] were also used for structural confirmation, using a Q-TOF setup. The fragmentation processes involving PHB-CD proton-charged parent ions proceed by two main pathways: the cleavage of the ester bonds through 1,4-hydrogen rearrangements (cleavage on the alkyl side) and cleavage of the 1,4-glycosidic bonds of glycoside units (Scheme 8). The daughter ions corresponding to the cleavage of the ester bonds were associated with the fragment observed at *m/z* 105, corresponding to the loss of protonated butyric acid from the end chains of PHB. Therefore, the authors concluded that the analyzed PHB-CD derivatives are random-substituted CDs with short oligoester chains.

The fragmentation patterns of ECD structures depend on the various substitution patterns and, in the case of ROP-synthesized PHB-CD, the substitution is random. Moreover, the presence of two fragmentation pathways complicates the MS/MS spectra, making their interpretation cumbersome. To better understand the MS/MS analysis, the fragmentation patterns of triacetyl-β-CD (commercial product with 21 acetyl units) and β-CD 3-hydroxybutyrate (custom-synthesized, with an average of 4 hydroxybutyric units per cyclodextrin) were compared [114]. In addition, proton- and sodium-charged species of both derivatives were examined considering that charge-induced fragmentation may provide certain selectivity at the level of cleaved bonds. Therefore, the proton-charged triacetyl-β-CD parent ion led to cleavage of the ester bonds and semiacetalic bonds, using an E_lab_ = 20 eV. On the other hand, in the fragmentation pattern of the sodium charged triacetyl-β-CD parent ion, the cleavages of the ester bonds are observed, while the glycosidic bond cleavages are less visible (E_lab_ = 120 eV). Such differences are caused by different charge–molecule interactions in the fragmented adduct. Probably, the bulky sodium cation interacts less with the oligosaccharide part of the molecule because of the steric effect produced by the full acetylation. In the case of partially substituted β-CD 3-hydroxybutyrate, the precursor ion chosen for fragmentation corresponded to a substitution degree of 5 units per β-CD. The fragmentation of the proton-charged species led to the expected results, with the semiacetalic bond cleavage as the main pathway due to the low number of substituent groups. On the other hand, the fragmentation performed on the sodium adducts revealed mainly the occurrence of the ester bond cleavage. Thus, the observed losses of 3-OH-butyric acid (a marker of the PHB OH chain end [120]) led to the conclusion that cyclodextrin is multiply substituted with 3-hydroxy-butyric acid instead of longer oligoester chains.

**Figure 11:**
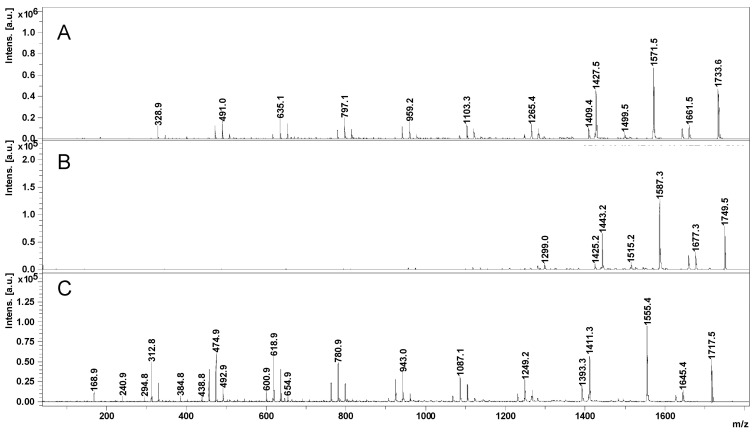
MALDI LID (TOF/TOF) of β-CDLA precursor ions: sodium- (**A**), potassium- (**B**), and lithium-charged (**C**) (reprinted with permission from [50]).

The fragmentation pattern of β-CDLA was studied using both soft ionization techniques ESI (CID in a QTOF setup) and MALDI MS/MS (LID—laser-induced dissociation on a TOF/TOF instrument) [50]. The LID fragmentation is available on some commercially available MALDI TOF/TOF instruments and consists of the possibility to obtain MS/MS spectra based on metastable fragmentation of MALDI-generated ions in milder energetic conditions than a CID process (MALDI TOF/TOF setup). For obtaining fragmentation spectra, such instruments work in LIFT mode as previously described for protein fragmentation [121]. The observed MS/MS fingerprints of β-CDLA obtained using CID (ESI QTOF—isolated monoisotopic parent ion) and LID (MALDI TOF/TOF—isolated isotopic cluster) were qualitatively similar, but the relative intensity of the fragment peaks was changed because of different fragmentation conditions. The fragmentation patterns were established using [CDLA + Na]^+^ parent ions for both types of fragmentation. Similar to other esterified CDs, the fragmentation can occur both at the level of glycoside units via cleavage of the semiacetalic bonds or at the level of oligolactide chains via cleavage of the ester bonds on acyl or alkyl sides. Furthermore, the fragmentation of CDLA with the same substitution degree was studied by MALDI LID (TOF/TOF) (Figure 11) using three cationization agents (NaI, LiI, KCl).

A similar fragmentation was observed for the sodium- and lithium-charged parent ions, involving both oligosaccharide and oligoester cleavages. On the other hand, the parent ions charged with potassium led mostly to cleavages of the ester bonds, revealing mainly the oligolactide side-chain fragmentation processes. Furthermore, the fragmentation patterns were correlated with the molecular mass of the parent ions, enabling the quantification of the average arm length (number of lactate monomer units per chain) grafted to the cyclodextrin.

In recent work, MALDI LID (TOF/TOF) analysis was employed to confirm the structure of minor peak series appearing in the MS spectra of CDLA. In principle, the isolation and characterization of these secondary products are rather difficult because of their low amount and also due to their high structural similarity to the main product. Thus, secondary β-CDLA products with different end groups were detected in the mass spectra of samples collected from various reaction mixtures [51]. Derivatives with acrylate, formate, or (methylenimino)butanoate end chains detected in mass spectra of samples collected from raw reaction mixtures (performed in DMSO, DMF, or NMP solvents) were subjected to MALDI LID (TOF/TOF) fragmentation studies. In addition to the main fragmentation patterns of the β-CD-oligolactide previously described, specific fragmentation patterns for each secondary derivative were detected in the MS/MS spectra. Therefore, for acrylated CDLA, a neutral loss of 54 Da (acrylate) was observed, confirming the structure of this derivative. Formate β-CD-oligolactide derivatives result as a consequence of DMF degradation in the reaction system, and the MS/MS fragmentation of the precursor ion containing 5 lactide units and one formate led to the neutral loss of 28 Da (CO) and 46 Da (HCOOH), confirming the attachment of the formate moiety. Similarly, NMP solvent also degrades, leading to secondary β-CDLA derivatives detected by MALDI MS, whose structure was confirmed by the investigation of the specific fragmentation patterns.

**Scheme 9:**
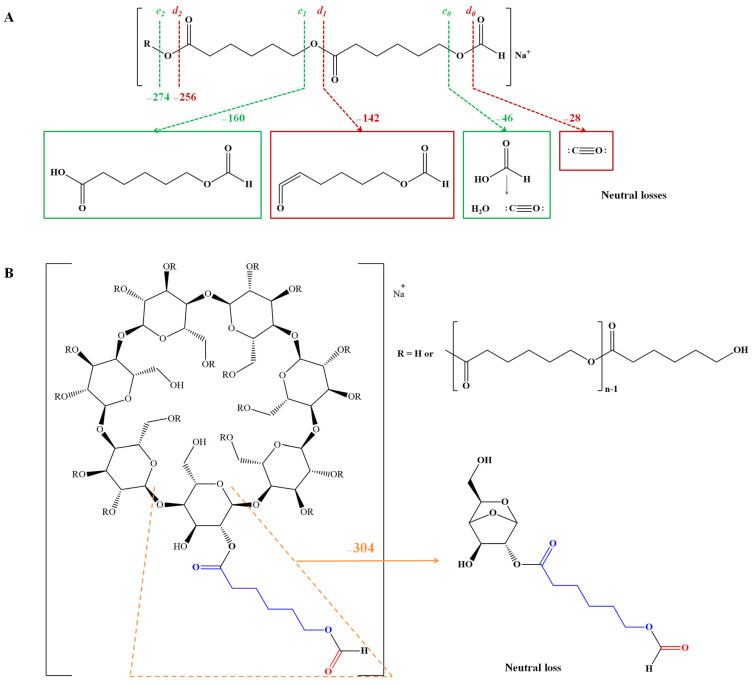
Fragmentation of CDCL derivatives at the level of oligocaprolactone chain and of the substituted β-CD (reprinted with permission from [46]).

Furthermore, MALDI LID (TOF/TOF) fragmentation studies were performed on CDCL products to establish their fragmentation pattern and to confirm the obtained results [46]. The fragmentation of CDCL derivatives proceeds similarly through cleavages of the ester bonds from the oligocaprolactone chains and of the semiacetalic bonds from β-CD (Scheme 9). Therefore, the precursor ion corresponding to β-CD substituted with 5 caprolactone units led to consecutive neutral losses of 114 Da (caprolactone monomer unit) through cleavage on the acyl sides, but higher intensity fragments corresponded to cleavages on the alkyl side of the ester bonds. Moreover, high-intensity fragmentation peaks were detected and associated with structures resulting from the cleavage of unsubstituted glycoside units or substituted with one or two 6-OH-hexanoate moieties. Side products such as formate-β-CDCL were detected in the reaction performed in DMF using MALDI MS, and their structure was confirmed by MS/MS analysis, similar to the previously discussed β-CDLA-formate derivatives.

## 4. Conclusions

The reviewed literature studies present a wide variety of mass spectrometry approaches aiming for accurate description of the complex cyclodextrin-esterified derivatives. Single-stage mass spectrometry experiments (both ESI and MALDI MS) are most often employed for structural confirmation and substitution degree measurement. Such studies were used for the structural elucidation of various cyclodextrin esterified with low-molecular-weight compounds such as carboxylic acids, acyl chlorides, anhydrides, vinyl esters, etc. Thus, the available mass spectrometry toolbox (ESI MS, LC-MS, and MALDI-TOF MS) has been employed for ECD evaluation and structural elucidation.

The MS techniques allowed the evaluation of the catalytic role of enzymes in the transesterification reactions involving cyclodextrins. The MALDI-TOF MS revealed the SD of the fatty-acid-functionalized cyclodextrins, and furthermore, LC MS showed that substitution’s selectivity may be partial for increased SD. In a distinct approach, the amphiphilic character of ECD modified with fatty acids was expressed as HLB (empirically established hydrophilic–lipophilic balance), as a function of the SD values calculated by MALDI-TOF MS. Moreover, the catalytic role of cyclodextrins themselves was investigated in the case of nitrophenyl ester transesterifications using MS methods.

The MS studies revealed the structure of the esterification products resulting from the ring opening of cyclic esters initiated by cyclodextrins. The ECD products were thoroughly investigated by MALDI-TOF MS, LC-MS, and fragmentation experiments. For cyclodextrin oligoesters (oligolactide, oligo-3-OH-butyrate, oligocaprolactone, and others), cyclodextrin substituents are structures with a variable number of monomer units per grafted chain, and the NMR spectra present an increased degree of complexity. In such situations, the LC or MALDI-TOF MS and NMR techniques completed each other to determine the average chain length and the average number of chains attached to the cyclodextrin. In addition, considering the possible bias when using the MALDI-TOF MS analysis for quantitative purposes, substitution degree calculations were confirmed using NMR spectroscopy, which allowed the determination of the kinetics of cyclic ester ring-opening oligomerization. In addition to the classical approach, mass spectrometry provides structural confirmation at the molecular level via gas phase fragmentation experiments (by collision-induced dissociation or laser-induced dissociation in MALDI-TOF setups) which may selectively access the oligosaccharide and the oligoester parts of the ECD architectures. Further insights into the structural assignment of the ECD isomers may be gained with the support of the ion mobility mass spectrometry method [122].

Overall, the employed organic and polymer synthesis using cyclodextrins as initiators/catalysts benefited from the mass spectrometry support, thus gaining significant advances in novel synthetic pathways and structural designs.

## Figures and Tables

**Table 1:** ECD obtained from modification with one molecule per substitution site and ECD mass spectrometry analysis.

CD Type	Esterification Agent	Substitution Site	Ionization	Observation	Ref.
β-CD	citric acid	OH2 or OH6	ESI—positive and negative mode	SD determination	[6]
β-CD	maleic acid itaconic acid	-	MALDI—positive mode	SD determination	[7]
6-O-tert-butyl- methylsilyl-β-CD	hexanoyl chloride hexanoyl anhydride	OH2 and OH3	ESI—positive mode	SD determination	[8]
6-O-tert-butyl- methylsilyl-α-, β-, γ-CD	hexanoyl chloride hexanoyl anhydride	OH2 and OH3	ESI—positive mode	SD determination	[9]
Heptakis(6-Br-6-deoxy)-β-CD	acetyl anhydride benzoyl bromide	OH2 and OH3	ESI—positive mode	structural confirmation (MS, MS/MS)	[10]
β-CD	capryloyl chloride lauroyl chloride palmitoyl chloride	random substitution	MALDI (DHB)	SD determination	[11]
β-CD	butanoyl chloride	-	MALDI (CHCA)	SD determination	[12]
β-CD	phenylacetyl chloride	random substitution	MALDI (DHB)	SD determination	[13]
β-CD	caproyl chloride	random substitution	MALDI (CHCA)	SD determination	[14]
β-CD	2-bromoisobutyryl bromide 2-methyl-2-(((undecyl-thio)carbonothioyl) thio) propanoic acid	substitution at OH6	MALDI (CHCA)	SD determination	[15]
γ-CD	acryloyl chloride	random substitution	MALDI	SD determination	[16]
α-CD, β-CD, γ-CD, maltoheptaose	succinic anhydride	fully substituted at OH 2, 3 and 6	MALDI (DHB)	Structural confirmation and MS detection (100 fmol)	[17]
α-CD, γ-CD	acetic anhydride	fully substituted at OH 2, 3 and 6	MALDI	Structural confirmation	[18]
β-CD	methacrylic anhydride	random substitution	MALDI	SD determination	[19]
β-CD	palmitoyl anhydride	OH2 and OH3	MALDI (IAA, THAP)	Structural confirmation	[20]
β-CD	3-((E)-dec-2-enyl)-di- hydrofuran-2,5-dione	monosubstituted at OH6	MALDI	Structural confirmation	[21]
γ-CD	succinic anhydride	random substitution	MALDI (CHCA)	SD determination	[22]
heptakis(2-O-benzyl-3-O-methyl)-β-CD	acetic anhydride	selective substitution at OH6	MALDI (THAP)	Structural confirmation	[23]
β-CD	1-methyl-2-(2′-carboxyethyl) maleic anhydride	-	MALDI	SD determination	[24]
β-CD	succinic anhydride based bis-pyridinium gemini surfactants	monosubstituted at OH6	ESI—positive mode	Accurate measurements, MS/MS	[25]
β-CD	divinyl dibutanoate divinyl dihexanoate divinyl didecanoate	monosubstituted at OH2	ESI—negative mode	SD determination	[26]
α-CD, β-CD, γ-CD	vinyl butyrate vinyl decanoate vinyl laurate	OH2 or random	MALDI (CHCA) ESI—positive mode	SD determination	[5]
β-CD	vinyl hexanoate vinyl decanoate	OH2	MALDI (DHB, CHCA)	SD determination	[27]
β-CD	vinyl butyric ester vinyl caproic ester vinyl caprylic ester vinyl capric ester vinyl lauric ester vinyl myristic ester	OH2	MALDI (DHB, CHCA)	SD determination	[28]
β-CD	vinyl acetate vinyl laurate	OH2	MALDI (CHCA)	SD determination	[29]
β-CD	vinyl butyric ester vinyl caproic ester vinyl caprylic ester vinyl capric ester vinyl lauric ester	OH2	ESI—positive mode	LC ESI MS analysis with LC ELSD confirmation	[30]
α-CD, β-CD, γ-CD	vinyl decanoate	OH2	MALDI (DHB)	SD determination, HLB-MALDI mesurement	[31]
α-CD, β-CD, γ-CD	vinyl laurate vinyl butyrate vinyl pivalate vinyl benzoate vinyl stearate	monosubstituted at OH2	MALDI (DHB)	Structural confirmation	[32]
β-CD	vinyl decanoate	OH2	MALDI	SD determination	[33]
β-CD	vinyl octanoate vinyl decanoate vinyl dodecanoate vinyl myristate	OH2	MALDI	SD determination	[34]
α-CD	m-nitrophenyl cinnamate	monosubstituted at OH2 or OH3	MALDI ESI—positive mode	Structural confirmation and proof for supramolecular interactions	[35]
α-CD, β-CD, γ-CD	4-nitrophenol-acrylate 4-nitrophenol-pent-4- enoate 4-nitrophenol-undec- 10-enoate	random substitution	MALDI (CHCA)	Structural confirmation—MS and MS/MS	[36]

**Table 2:** ECDs obtained by ring opening of cyclic esters and OECD mass spectrometry analysis.

CD Type	Esterification Agent	Substitution Site	Ionization	Observation	Ref.
α-CD, β-CD, γ-CD, mono-2-O-(6-benzoxy-pentanoyl)-β-CD	β-butyrolactone δ-valerolactone ε-caprolactone	OH2	MALDI (DHB)	Structural confirmation	[37,38,39]
cinnamoyl-α-CD	δ-valerolactone	OH2	MALDI	Structural confirmation	[40]
β-CD	β-butyrolactone	random substitution	ESI—positive mode	structural confirmation (MS, MS/MS), LC MS, number of oligoester arms—MS and NMR	[41]
dihydroxyl per-O-benzyl-β-CD	rac-Lactide rac-butyrolactone	OH6	MALDI (CHCA)	Structural confirmation	[42]
β-CD	β-butyrolactone	mainly at OH6	ESI—positive mode	Structural confirmation—LC MS, number of oligoester arms—MS and NMR	[43]
β-CD	ε-caprolactone	-	MALDI	Structural confirmation of CD derivatives and side products	[44]
β-CD	D,L-lactide L-lactide ε-caprolactone	random substitution	MALDI (DHB, HABA)	Structural confirmation	[45]
β-CD	ε-caprolactone	random substitution	MALDI (DHB, CHCA)	Structural confirmation (MS, MS/MS), MS kinetics with NMR confirmation, number of oligoester arms—MS and NMR	[46]
β-CD	lactide	OH6	ESI—positive mode	Structural confirmation	[47]
Dihydroxyl per-O-benzyl-β-CD	D,L-lactide	OH6	MALDI (dithranol)	Structural confirmation	[48]
α-CD, β-CD, γ-CD	L-lactide	OH6	MALDI (CHCA) ESI—positive mode	Structural confirmation, LC with ELSD detection and offline MALDI MS, number of oligoester arms—MS and NMR	[49]
β-CD	L-lactide	OH6	MALDI (CHCA) ESI—positive mode	Structural confirmation and oligoester arms length by MS/MS	[50,58]
β-CD	D,L-lactide	OH6	MALDI (DHB, CHCA)	Structural confirmation (MS, MS/MS), MS kinetics with NMR confirmation, number of oligoester arms—MS and NMR	[51]

## Data Availability

Not applicable.
